# Effects of Sub-lethal Concentrations of Silver Nanoparticles on a Simulated Intestinal Prokaryotic–Eukaryotic Interface

**DOI:** 10.3389/fmicb.2017.02698

**Published:** 2018-01-15

**Authors:** Elisa Garuglieri, Erika Meroni, Cristina Cattò, Federica Villa, Francesca Cappitelli, Daniela Erba

**Affiliations:** Department of Food, Environmental and Nutritional Sciences, Università degli Studi di Milano, Milan, Italy

**Keywords:** silver nanoparticles, intestinal biofilm, Caco-2 cells, prokaryotic–eukaryotic interface, sub-lethal concentrations

## Abstract

Nanotechnology applications are expected to bring a range of benefits to the food sector, aiming to provide better quality and conservation. In this research, the physiological response of both an *Escherichia coli* mono-species biofilm and Caco-2 intestinal cells to sub-lethal concentrations of silver nanoparticles (AgNPs) has been investigated. In order to simulate the anaerobic and aerobic compartments required for bacteria and intestinal cells growth, a simplified semi-batch model based on a transwell permeable support was developed. Interaction between the two compartments was obtained by exposing Caco-2 intestinal cells to the metabolites secreted by *E. coli* biofilm after its exposure to AgNPs. To the best of the authors’ knowledge, this study is the first to investigate the effect of AgNPs on Caco-2 cells that takes into consideration previous AgNP-intestinal biofilm interactions, and at concentrations mimicking real human exposure. Our data show that 1 μg/mL AgNPs in anaerobic conditions (i) promote biofilm formation up to 2.3 ± 0.3 fold in the first 72 h of treatment; (ii) increase reactive oxygen species (ROS) production to 84 ± 21% and change the physiological status of microbial cells after 96 h of treatment; (iii) seriously affect a 72-h old established biofilm, increasing the level of oxidative stress to 86 ± 21%. Moreover, the results indicate that oxygen renders the biofilm more adequate to counteract AgNP effects. Comet assays on Caco-2 cells demonstrated a protective role of biofilm against the genotoxic effect of 1 μg/mL AgNPs on intestinal epithelial cells.

## Introduction

Because of their unique physico-chemical properties, nanoparticles (NPs) are widely used in the agri-food industry as agrochemicals for controlling pests and the delivery of active ingredients, nanosized ingredients, and additives (Chaudhry et al., 2008; Polo et al., 2011; Chen et al., 2016; Grillo et al., 2016). Another interesting and growing nanomaterial application is for functionalizing food processing surfaces and for packaging, mostly to improve mechanical and antimicrobial properties (Dainelli et al., 2008).

Given these widespread applications, NP exposure represents a potential toxicity risk for human health. Of special interest is the effect of NPs on human gut microbiota, considering the range of consumer goods that can be intentionally, or accidentally ingested. However, most of the literature regarding NP effects on human health concern lung cell *in vitro* cultures (Geary et al., 2016). Only a few animal studies have been made on NP gastrointestinal intake and impact, insufficient to allow a clear safety assessment of ingested NPs (Tran and Chaudhry, 2010; Bergin and Witzmann, 2013; Jones et al., 2015; Pietroiusti et al., 2016) or a better understanding of their impact on the intestinal ecosystem (Fröhlich and Fröhlich, 2016).

A major reason for the scarcity of relevant literature on NP effect on the gut ecosystem is related to the lack of effective and simplified model systems for studying the nature of these complex interactions. Furthermore, most of the studies were conducted with relatively high (bactericidal) NP concentrations, and the effect of sub-lethal exposure (low concentrations) is poorly understood. Thus, there is a critical gap in current knowledge, low NP concentrations (rather than high), following dilution and dispersion, being expected to predominate in both the food chain and the gastrointestinal system (Benn and Westerhoff, 2008; Colman et al., 2013).

In addition, there is growing recognition that intestinal microflora exists as biofilm differing in characteristics from their planktonic counterpart (Donelli et al., 2012). Despite the evidence of human microbiome as a biofilm on gut mucosa (Donelli et al., 2012; Donaldson et al., 2016), the complexity of such intestinal biofilm and its interaction with low NP concentrations (the actual human consumption) and epithelial intestinal cells is still largely unknown.

In order to lay the foundation for understanding the complex interplay among NPs, gut biofilm and its host, the physiological response of a simplified system composed by *Escherichia coli* mono-species biofilm and Caco-2 intestinal cells exposed to sub-lethal concentrations of silver nanoparticles (AgNPs) was investigated. *E. coli* was chosen as representative of the gut biofilm, being the best characterized model microorganism and the most frequent commensal aero-anaerobic Gram-negative bacillus of the vertebrate gut (Tenaillon et al., 2010). AgNPs are the most commonly used metal nanoparticles in different applications (Zhang et al., 2016a).

To simulate the anaerobic and aerobic compartments required for bacteria and intestinal cells growth, a semi-batch model based on a transwell permeable support was developed. Interaction between the two compartments was obtained by exposing Caco-2 intestinal cells to the metabolites secreted by *E. coli* biofilm after its exposure to AgNPs.

To the best of the authors’ knowledge, this study is the first to investigate the effect of AgNPs on Caco-2 cells that takes into consideration previous AgNP-intestinal biofilm interactions, and at concentrations mimicking real human exposure.

## Materials and Methods

### Silver Nanoparticles

The AgNPs (BioPure, NanoComposix, San Diego, CA, United States, 10 nm, 1 mg/mL in 2 mM citrate buffer) were stored at 4°C and directly diluted to 1 μg/mL in bidistilled water or culture media just before use in the experiments. According to the supplier, purchased AgNPs have a diameter of 8.5 ± 1.7 nm (JEOL 1010 Transmission Electron Microscope), a hydrodynamic diameter smaller than 20 nm and a negative zeta potential of -27.3 Mv (Malvern Zetasizer Nano ZS). AgNP size was assessed by transmission electron microscopy (TEM) by our team in previous work and was found to be 14 ± 0.3 nm (Garuglieri et al., 2016).

### Gut Interactive Model

**FIGURE 1:**
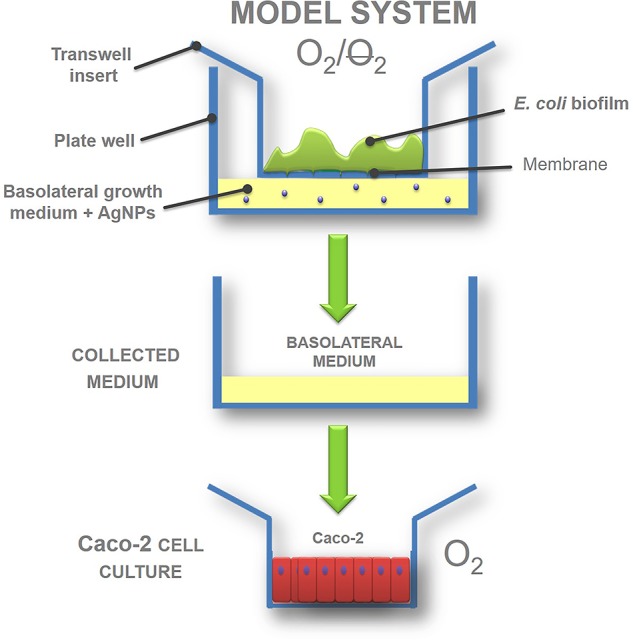
Gut interactive *in vitro* model outline: transwell biofilm culture exposed to silver nanoparticles (AgNPs) and *in vitro* system workflow.

**Figure 1** shows the experimental model used to reproduce an interactive gut ecosystem. Indeed, the effect of AgNPs on biofilm and on Caco-2 cells was analyzed separately. The biofilm was grown in anaerobic conditions (see section “Effect of AgNPs on Biofilm”), mimicking the conditions of the intestine lumen, while the Caco-2 cells, which need oxygen to live, were grown under aerobic conditions (see section “Effect of AgNPs on Caco-2 Cells”). The AgNP response was studied (i) in the early stages and (ii) on established *E. coli* biofilm. The interaction of the AgNPs, biofilm and Caco-2 cells was assessed by putting into contact the biofilm basolateral media and the Caco-2 cells.

Due the lack of information about the interaction of AgNPs with anaerobic biofilm (Yang et al., 2013; Gitipour et al., 2016), and to better understand the effect of oxygen in the bacterial AgNP response, biofilm was also grown in aerobic conditions.

The experiments were performed in the presence of 1 μg/mL AgNPs, the chosen representative of food-related, sub-lethal concentrations of human exposure (Gottschalk et al., 2013; Kuorwel et al., 2015; Fröhlich and Fröhlich, 2016).

### Effect of AgNPs on Biofilm

#### Bacterial Strains and Planktonic Growth Conditions

The well characterized *E. coli* MG 1655 and green fluorescent protein (GFP)-*E. coli* MG 1655 were stored at -80°C in phosphate-buffered saline solutions (PBS, Medicago AB, Uppsala, Sweden) containing 20% glycerol. Both microorganisms were routinely cultured in liquid Tryptic Soy Broth medium (TSB, Conda, Italy) with an addition of 100 mg/L ampicillin (Sigma–Aldrich) for the GFP strain, at 37°C in aerobic or anaerobic atmosphere. Anaerobic experiments were performed in an anaerobic box (Forma Scientific, Marietta, OH, United States) under N_2_:H_2_:CO_2_ atmosphere (85/10/5, v/v) using TSB pre-reduced in anaerobic conditions for 24 h before experiments began. In previous research, TSB was chosen as the best nutritive medium that could guarantee the satisfactory stability of dispersed AgNPs against time (Garuglieri et al., 2016).

#### Transwell Biofilm Cultures

A sterile polycarbonate membrane (PC, Whatman Nuclepore, diameter 2.5 cm, pore diameter 0.2 μm) was carefully placed on a sterile TSA plate and inoculated at its center with 50 μL of an overnight TSB culture of *E. coli*/GFP-*E. coli* grown at 37°C in aerobic/anaerobic conditions. Inocula were normalized through optical density (OD) measurements at 600 nm with a JENWAY 7315 Spectrophotometer, to obtain a final concentration of 10^8^ cells/mL. The membrane was left on the agar plate until the inoculum had dried completely, after which it was carefully transferred to inside the transwell (ThinCert^TM^ Cell Culture Inserts with translucent PET membrane – Greiner bio-one), inlaid in a six well culture plate (Greiner bio-one). One mL of TSB medium (pre-reduced if working in anaerobic conditions) was added to the plate well (basolateral compartment). Biofilm was grown at 37°C in both aerobic and anaerobic conditions, and to guarantee continuous growth, the transwells were transferred every 24 h to new plate wells with fresh TSB. Basolateral media were collected every 24 h and stored at -80°C to be used in further experiments with Caco-2 cells (aerobic part of the model).

#### Biofilm Exposure to AgNPs

Biofilm of *E. coli* was cultured using transwell setups as previously described and AgNP exposure was performed in two different ways. In the first assay, the biofilm was grown for 96 h with the addition of 1 μg/mL AgNPs dispersed in the basolateral medium (effect on biofilm formation); in the second assay, the AgNPs were added for 24 h directly onto the surface of an established biofilm, pre-grown for 72 h without AgNPs (effect on established biofilm). Control samples without AgNPs were run simultaneously. Every 24 h, PC membranes with adherent biofilm were removed from the transwell setups, transferred to tubes containing 1 mL of sterile PBS, vortexed for 1 min, and sonicated for 3 min in a water bath (Sonica Ultrasonic Cleaner, Soltec, Milan, Italy). Using this procedure, all the cells were dislodged from the membranes, and clumps of cells were broken apart. Serial dilutions of 10 μL of the cell suspension were plated in triplicate on Tryptic Soy Agar (TSA, Fisher Scientific) to perform a plate count viability assay as reported by Gambino et al. (2015). All plates were incubated overnight at 30°C. Colony forming units (CFUs) were determined by standard colony counting method. Experiments were conducted in triplicate under both anaerobic and aerobic conditions.

#### Biofilm Sectioning and Imaging by Confocal Laser Scanning Microscopy

*Escherichia coli*-GFP biofilm was grown in triplicate under two different AgNP exposure times, as described in Sections “Transwell Biofilm Cultures” and “Biofilm Exposure to AgNPs.” At 96 h, biofilm adhering to the PC membrane was carefully covered with a layer of Killik cryostat embedding medium (Bio-Optica, Milan, Italy) and placed at -80°C until completely frozen. Frozen samples were sectioned at -19°C using a Leica CM1850 cryostat, and the 5 μm thick cryosections were mounted on Superfrost Plus microscope slides (Fisher Scientific). Samples were observed using a Nikon Eclipse E800 microscope with a 10 or 20X dry objective. The sections were viewed both in bright-field and in epifluorescence mode.

The software ImageJ (Schneider et al., 2012) was used to perform the image analysis and to calculate the biofilm thickness of the control and treated samples. More than five images per sample were taken for microscope analysis. For each picture, the biofilm thickness was measured at three different locations randomly selected along the profile. These measurements were used to calculate the average thickness and the associated standard deviation (SD).

The percentage of green GFP positive signals in the biofilm sections was assessed using the standard tools ‘segmentation and quantification of cellular structures’ of ImageJ software. Average intensity measurements of fluorescence were collected at the periphery and center of the biofilm clusters. The regions were square dimensions of 875 μm^2^ therefore the total areas analyzed at the periphery and center of each cluster were equal. Intensity values were also normalized by dividing the fluorescence intensity of the AgNP treated samples (I) by the fluorescence intensity values of the control (I_0_) obtained at the same location.

#### Level of Oxidative Stress in Biofilm Cells

The level of oxidative stress in *E. coli* biofilms was assessed using the 2,7-dichlorofluorescein-diacetate (H_2_DCFDA, Sigma–Aldrich, St. Louis, MO, United States) assay (Jakubowski and Bartosz, 2000). Biofilm samples of *E. coli* were cultured using transwell setups and exposed to two AgNP treatments, as reported in Sections “Transwell Biofilm Cultures” and “Biofilm Exposure to AgNPs.” Every 24 h, three PC membranes with adhered biofilm were removed from the transwell insert, and the biofilms was recovered as in Section “Biofilm Exposure to AgNPs.” The obtained cellular suspensions were washed twice with PBS (13000 rpm, 15 min) and resuspended in 50 mM PBS. The cells were then broken using glass beads (0.1 μm diameter) and the Precellys 24 (Bertin Technologies, France) bead–beater device with a beating profile of 3 s × 30 s at 6500 rpm. After centrifugation (13000 rpm, 15 min), 750 μL of supernatant were incubated with 4 μL of 10 M H_2_DCFDA at 30°C for 30 min. The solution was homogeneously divided into three wells of 96 wells black microtiter plates (Greiner bio-one). The relative fluorescence correlated to the reactive oxygen species (ROS) amount was measured with excitation at 490 nm and emission at 519 nm, using the Infinite F200 PRO microtiter plates reader (TECAN, Mannedorf, Switzerland). The relative fluorescence was normalized against the number of cells, obtained by a viable count of initial cell suspensions. Experiments were conducted in triplicate, in both aerobic and anaerobic conditions.

### Effect of AgNPs on Caco-2 Cells

#### Cell Line

Human Caco-2 cells were obtained from the European Collection of Animal Cell Cultures (United Kingdom). The cells were cultured in Dulbecco’s Modified Eagle’s Medium (DMEM, Sigma–Aldrich) supplemented with 10% heat inactivated (30 min at 56°C) fetal bovin serum, 2 mM L-glutamine, 100 U/ml penicillin, 100 mg/ml streptomycin, 0.1 mM non-essential amino acids, in an incubator with an atmosphere of 95% air and 5% carbon dioxide. The culture medium was routinely changed every 2 days, and always the day before exposure to AgNPs. All cell culture reagents were purchased from Sigma–Aldrich (St. Louis, MO, United States) and chemicals from Merck (Darmstadt, Germany).

#### Cell Line Maintenance and Subculturing

When cells reached a subculturing density of 70% confluence, they were detached by means of trypsinization: the medium was removed from the flask (75 cm^2^), and the cells were washed and treated with 2.5 mL of fresh trypsin–EDTA solution in the incubator. The trypsin action, lasting 4 min, was arrested by the addition of 4 mL of complete medium. The cell suspension was then transferred to a 15 mL tube and centrifuged 5 min at 2300 rpm. After removing the supernatant, the cell pellet was resuspended in complete medium and seeded at 10^4^ cells/cm^2^ (Natoli et al., 2012).

#### Cell Differentiation

For the differentiation experiments, the cells were seeded on 24-well plates (Cellstar, Greiner) at a density of 10^5^ cells per well and, after confluence, maintained for 10 days in complete medium; the medium was changed three times a week (Sambuy et al., 2005).

#### Cytotoxicity and Genotoxicity Analysis

After being cultured 10 days, the differentiated Caco-2 cell monolayers were incubated with: (i) TSB without AgNPs (TSB), (ii) TSB with 1 μg/mL AgNPs (TNP), (iii) all the basolateral media from the anaerobic biofilm culture grown without (C24, C48, and C72) and with AgNPs (NP24, NP48, and NP72) for 24, 48, 72 h, diluted 1:1 with the medium. The treatments lasted for 1 h at 37°C, in an incubator with 95% humidity and 5% CO_2_. Each treatment was performed in triplicate, and negative (PBS) and positive (H_2_O_2_, 50 μM) controls were included in each experimental batch to verify the reliability of the Comet assay procedure (Venturi et al., 1997).

After incubation, the cells were detached by trypsinization and an aliquot of this cell suspension was used to assess the cytotoxicity of the treatment, by measuring cell viability with the Trypan Blue exclusion test (expressed as percentage of viable cells) (Strober, 2001). Trypan Blue is a dye that selectively colors only dead cells; live cells have an intact membrane that does not allow the dye to penetrate the cytoplasm, whereas in dead cells the dye penetrates easily, thus distinguishing them from the others.

Another aliquot of cell suspension was used for the Comet assay to assess the genotoxicity of the treatments. Briefly, the cells were centrifuged (10000 rpm, for 15 s), re-suspended in 1% low-melting point agarose, and spread on a microscope slide previously covered with a 1% normal-melting point agarose layer. Embedded cells were lysed, DNA was allowed to unwind in electrophoresis buffer (pH 10) and then electrophoresis was performed at 25 V and 300 mA for 20 min. After this step, the slides were immersed in a neutralization buffer for 15 min, stained with ethidium bromide and analyzed using a fluorescence microscope (BX60 Olympus, Japan) equipped with Image-Pro Plus software (Immagini & Computer, Milan, Italy). The formation of a comet-like tail implies the presence of a damaged DNA single strand: the length of the tail increases with the extent of DNA damage (AshaRani et al., 2009). Fifty images were analyzed for each slide and tail moment registered: DNA damage was expressed as percentage of DNA in the tail (Tice et al., 2000).

### Statistical Analysis

To evaluate statistically significant differences among the samples, the analysis of variance test (ANOVA) was performed using MATLAB software (The MathWorks, Inc., Natick, MA, United States). The ANOVA analysis was carried out after verifying whether the data satisfied the assumptions of (i) independence, (ii) normal distribution, and (iii) homogeneity of variance. Tukey’s honestly significant different test (HSD) was used for pair wise comparison to determine data significance. Differences were considered significant for *p* < 0.05.

## Results

### AgNPs Effects on Biofilms

**FIGURE 2:**
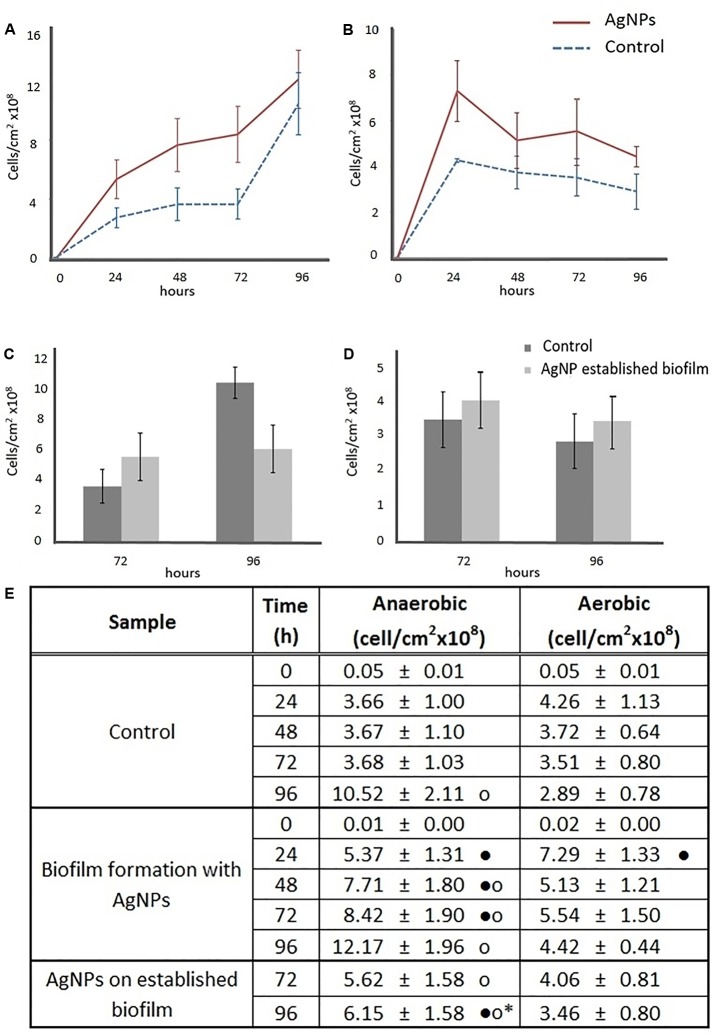
Effects of AgNPs on *Escherichia coli* biofilm formation **(A,B)** and on a 72 h-old *E. coli* established biofilm **(C,D)**. Growth curves were based on viable counts in presence of 0 (control) and 1 μg/mL AgNPs in anaerobic **(A,C)** and aerobic **(B,D)** conditions. Data in the table **(E)** represent the means ± standard deviation (SD) of three independent measurements. Dots provide the graphical representation for *post hoc* comparisons. According to *post hoc* analysis [Tukey’s honestly significant different test (HSD), *p* < 0.05], means sharing ∙ show statistical difference to the relative control, and anaerobic means sharing o show statistical difference to the aerobic counterparts. Means sharing • show statistical difference to the biofilm formation in presence of AgNPs counterparts.

**Figures 2A,E** show the number of cells in biofilm grown in anaerobic conditions with and without 1 μg/mL AgNPs in the basolateral media. The data clearly showed a significant increase in the number of adhered cells in biofilm treated with AgNPs, compared to the control, up to 72 h (24 h: 1.5 ± 0.4 fold; 48 h: 2.1 ± 0.4 fold; 72 h: 2.3 ± 0.3 fold). On the contrary, after 96 h of incubation with AgNPs, the biofilm reached a number of cells comparable to the control. In aerobic conditions, the treated biofilm showed an increase of 1.7 ± 0.3 fold in the number of adhered cells with respect to the control only in the first 24 h (**Figures 2B,E**). However, after 48 h incubation, both the control and the treated biofilm reached a stationary phase, with a similar number of cells. Comparing anaerobic and aerobic data, both the treated (48, 72, 96 h) and the control (96 h) biofilm showed a higher number of adhered cells in the absence of oxygen (**Figure 2E**).

In a second experiment, AgNPs were added for 24 h directly onto the surface of established biofilm pre-grown for 72 h without AgNPs. In anaerobic conditions, after the AgNP addition (96 h), the number of adhered cells on the biofilm was lower (-41.5 ± 7.5%) than on the untreated control biofilm (**Figures 2C,E**). Indeed, the number of viable cells in the treated biofilm did not change before (72 h) or after (96 h) AgNP exposure, however, for the same time interval, the number of cells in the control biofilms increased. Under aerobic conditions, there was no statistical differences in the number of adhered cells in either the control or the AgNP treated biofilm (**Figures 2D,E**), but for the established biofilm treated with AgNPs there was a higher number of adhered cells in the absence of oxygen than in the presence of oxygen (**Figure 2E**).

### Biofilm Sectioning and Imaging by Confocal Laser Scanning Microscopy

**FIGURE 3:**
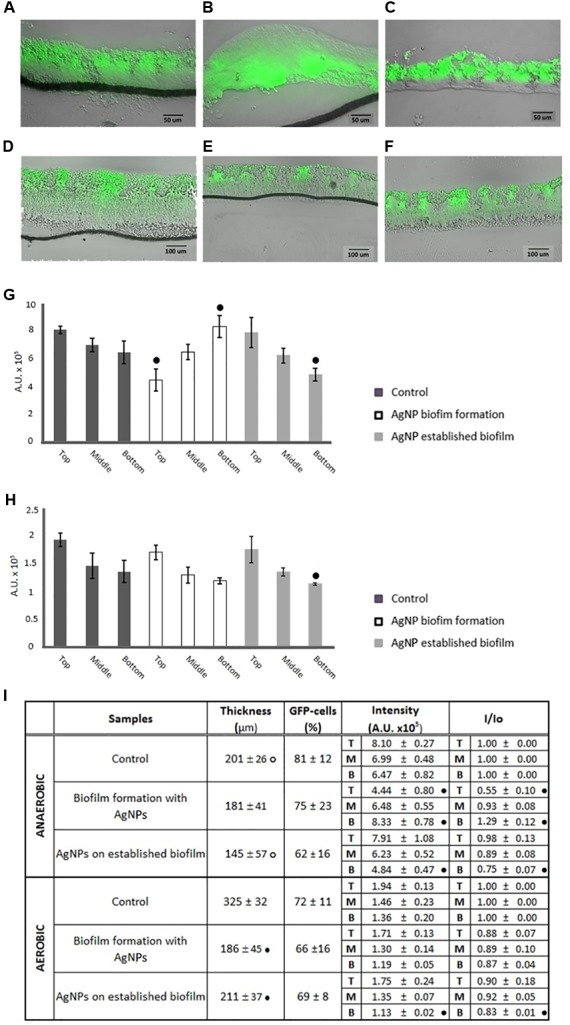
Merged bright-field and fluorescence images of representative 5-μm-thick cryosections of green fluorescent protein (GFP)-*E. coli* biofilms under both anaerobic **(A–C)** and aerobic **(D–F)** conditions. Images were collected after 96 h incubation under the following conditions: control (**A,D**, grown for 96 h in absence of AgNPs), AgNP exposure during biofilm formation (**B,D**, grown for 96 h in presence of 1 μg/mL AgNPs), and AgNP exposure on established biofilm (**C,F**, grown for 72 h in absence of AgNPs and then treated with 1 μg/mL AgNPs for 24 h). **(G)** Anaerobic condition and **(H)** aerobic condition show values of fluorescence intensity ± SD of GFP-positive cells from different positions within the biofilm thickness: top, middle, and bottom; different letters indicate statistical differences between top, middle, bottom within the same biofilm section (Tukey’s HSD, *p* < 0.05). In table **(I)** biofilm thickness, percentage of GFP-positive cells, values of fluorescence intensity and fluorescence intensity normalized to the controls (I/Io) at the top (T), middle (M), and bottom (B) of the biofilm, in both anaerobic and aerobic conditions, are shown; data represent the means ± standard deviation (SD) of at least three independent experiments; according to *post hoc* analysis (Tukey’s HSD, *p* < 0.05), means sharing ∙ show statistical difference to the control and anaerobic means sharing • show statistical difference to the aerobic counterparts.

Under anaerobic conditions, cryosectioning combined with microscopy revealed no statistically differences in biofilm thickness between the control and the samples grown with 1 μg/mL AgNPs (**Figures 3A,B,I**) or exposed for 24 h to the same AgNPs concentrations after pre-grown without AgNPs for 72 h (**Figures 3A,C,I**). By contrast, in aerobic conditions, biofilms treated with AgNPs showed a reduced thickness with respect to the control. Indeed, biofilm grown in the presence of AgNPs for 96 h resulted in a 42.8 ± 11.1% decrease in thickness with respect to the negative control (**Figures 3D,E,I**), while when AgNPs were added for 24 h directly onto the surface of a pre-grown biofilm, a 35.1 ± 7.0% decrease in thickness was shown (**Figures 3D,F,I**).

Comparing the samples in the different atmospheres: in anaerobic and aerobic conditions biofilm grown in the presence of AgNPs showed the same thickness (**Figure 3I**). However, in an anaerobic environment the biofilm was thicker than in aerobic conditions when the AgNPs were loaded later onto the surface of biofilm pre-grown in the absence of NPs (**Figure 3I**). Interestingly, control biofilm grown in anaerobic (**Figure 3D**) conditions was thicker than that grown in the presence of oxygen (**Figure 3I**).

After 96 h growth under AgNP exposure in anaerobic conditions, the analysis of pictures also revealed that 74 ± 23% of cells were scored as GFP-positive, with a value statistically similar to that of the control (**Figure 3I**). A similar number of GFP-positive cells was found in the 72 h-old established biofilm after the addition of 1 μg/mL AgNPs for 24 h (**Figure 3I**). In aerobic conditions, no statistical difference in the % of GFP-positive cells was recorded after AgNP treatment (**Figure 3I**). Moreover, the aerobic biofilm showed a similar percentage of GFP-positive cells to its anaerobic counterpart (**Figure 3I**).

As shown in **Figure 3**, the GFP-positive cells displayed different distribution patterns within the biofilms. Indeed, in the control samples, after 96 h of growth under both anaerobic and aerobic conditions, the GFP-positive population was predominantly located along the biofilm-air interface (top of the biofilm) while cells in the middle and at the bottom of the biofilm were less active with respect to the GFP protein expression (**Figures 3G–I**). On the contrary, when biofilm was grown in anaerobic conditions with AgNPs, GFP-positive cells displayed an inverted distribution. Indeed, the GFP positive cells appeared seriously reduced at the apical part of the biofilm (-45 ± 10%), while at the bottom of the biofilm, at the interface with the membrane, fluorescence appeared to be increased 30 ± 12% (**Figures 3G,I**). Biofilm pre-grown in an anaerobic environment without NPs and further treated with AgNPs showed the same pattern distribution of the control with a predominance of active cells in the upper part of the biofilm section (**Figures 3G,I**).

Under aerobic conditions, biofilm grown with AgNPs showed the same GFP distribution pattern as the corresponding control, while an 18 ± 1% reduction of active cells at the bottom of the biofilm was observed when the biofilm had been treated with AgNPs after 72 h pre-growth.

### Level of Oxidative Stress in Biofilm under AgNP Exposure

**FIGURE 4:**
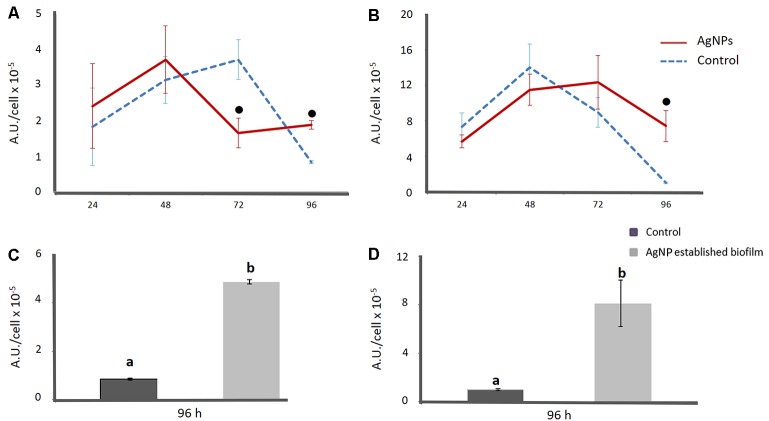
Level of oxidative stress within the *E. coli* biofilm under both anaerobic **(A–C)** and aerobic **(B–D)** conditions. Data are referred to biofilm grown for 96 h in presence and absence of AgNPs **(A,B)** and to a 72-old established biofilm grown in absence of AgNPs and further treated with AgNPs for 24 h **(C,D)**. Data represent the means ± SD of three independent measurements. According to *post hoc* analysis (Tukey’s HSD, *p* < 0.05), means sharing the same letter are not significantly different from each other and means sharing • showed significant difference in respect to control.

In order to better understand the effect of AgNPs on biofilm formation, the level of oxidative stress within biofilm was evaluated. As shown in **Figure 4A**, up to the first 48 h under anaerobic conditions the oxidative stress of biofilm grown in the presence of 1 μg/mL AgNPs was statistically similar to that in the control. At 72 h, there was a significant decrease in the ROS level, -55 ± 7% was recorded, while at 96 h the data revealed an opposite trend with a level of ROS 55 ± 4% higher in treated biofilm with respect to untreated. The addition of AgNPs to pre-grown biofilm further promoted the increase in ROS, with a percentage of ROS 82 ± 1.5% higher than in the control (**Figure 4C**).

In aerobic conditions, the oxidative stress of biofilm grown for 96 h with AgNPs was not statistically different to the control until 72 h (**Figure 4B**). After 96 h of incubation with AgNPs, a sudden decrease in the level of oxidative stress was recorded in both the control and treated biofilm (**Figure 4B**). However, in the treated biofilm, the level of oxidative stress was 84 ± 21% higher than in the control. The addition of AgNPs to pre-grown biofilm also increased the level of ROS (86 ± 21%), with values comparable to those obtained by growing biofilm with AgNPs for 96 h (**Figure 4D**).

Comparing results obtained in anaerobic and aerobic environments, the level of oxidative stress was always lower in anaerobic conditions than in aerobic ones.

### Cytotoxicity and Genotoxicity

**FIGURE 5:**
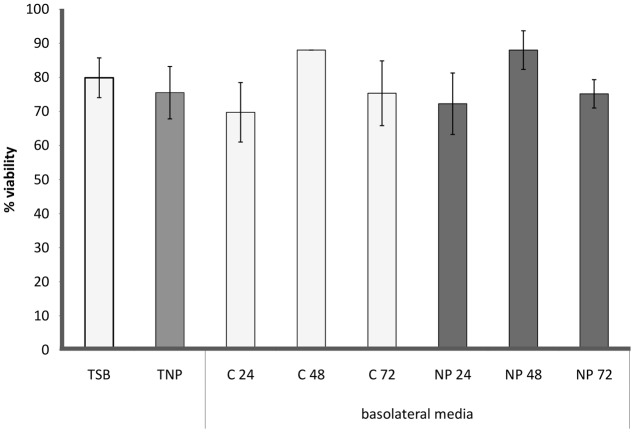
Cell viability of differentiated Caco-2 cells incubated with TSB (TSB), TSB with 1 μg/mL AgNPs (TNP), all the basolateral media from the anaerobic biofilm grown without (C24, C48, C72) and with AgNPs (NP24, NP28, NP72).

In the cytotoxicity assay neither TNP nor basolateral media samples were found to exert cytotoxic effects on Caco-2 cells (**Figure 5**). The viability of the treated cells was consistently >70% and not significantly different from that of cells treated with TSB; consequently, all treated cell suspensions were processed for Comet Assay.

**FIGURE 6:**
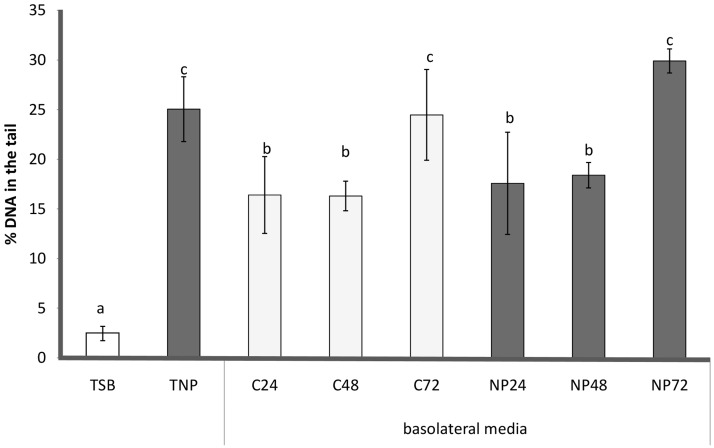
Comet Assay: genotoxicity of different treatments, expressed as % of DNA in the tail (mean ± SD). Caco-2 cells were incubated with TSB (TSB), TSB with 1 μg/mL AgNPs (TNP), all the basolateral media from the anaerobic biofilm culture grown without (C24, C48, C72) and with AgNPs (NP24, NP48, NP72). Data not sharing common letter are significantly different, *p* < 0.05.

DNA oxidative damage was assessed using the Comet assay (**Figure 6**). TSB treatment resulted in very little damage (<5%), whilst in cells treated with TNP, the DNA oxidative damage significantly increased, about 10-fold. All the basolateral media treatments generated moderate oxidative DNA damage (<30%). Cells treated with basolateral media collected at 24 and 48 h, from both control (C24, C48) and chronic (NP24 and NP48) samples, were statistically similar to each other and showed a significant increase in DNA oxidative damage (16–19%) with respect to TSB. Moreover, cells treated with basolateral media collected at 72 h from both control and chronic samples (C72, NP72) were statistically similar to each other and showed values of DNA oxidative damage (about 25%) statistically higher than those found for 24 and 48 h samples.

Indeed, DNA damage was not significantly affected by AgNP presence in basolateral media collected from chronic samples with respect to those collected from control samples at the same time. Therefore, it was observed that until 48 h DNA oxidative damage of chronic basolateral media was significantly less than the TNP treatment.

## Discussion

In our study, intestinal *E. coli* biofilm was grown in a transwell device under anaerobic conditions, and the interaction between 1 μg/mL AgNPs, biofilm and Caco-2 cells was assessed by putting into contact the basolateral media containing the biofilm metabolites with Caco-2 cells grown with oxygen. To date, many studies have focused on the direct toxic effects of NPs on exposed cells and organisms, which may not reflect the real situation observed *in vivo* (Fröhlich and Fröhlich, 2016). In order to mimic the real intestinal epithelium exposure conditions, Caco-2 cells were treated with basolateral media from the microbial barrier, in which metabolites, produced by biofilm after its interaction with AgNPs, are present. In addition, experiments were conducted using a concentration of AgNPs (1 μg/mL) representative of their actual food-related ingestion (Gottschalk et al., 2013; Kuorwel et al., 2015; Fröhlich and Fröhlich, 2016).

A variety of *in vitro* models have been proposed to simulate and study the microbiome of the human gastrointestinal system environment. These can be divided into (i) batch and (ii) dynamic systems (Marzorati et al., 2014). Batch systems usually consist of small reactor vessels or test tubes modeling one single segment of the gastrointestinal tract. Batch experiments offer a very easy and flexible screening tool (Venema and Van den Abbeele, 2013), but they do tend to over-simplify the complexity of the gastrointestinal process (Marzorati et al., 2014). Moreover, they are far from physiological conditions, and suffer, for example, from the accumulation of microbial metabolites, thus inhibiting further microbial activity so that incubation usually cannot be extended to periods over 24 h (Venema and Van den Abbeele, 2013). Instead, continuous dynamic models allow in-depth studies of the biological processes in the gut under more representative physiological conditions (Venema and Van den Abbeele, 2013; Marzorati et al., 2014). Some dynamic systems, e.g., SHIME (Van den Abbeele et al., 2010), EnteroMix (Makivuokko et al., 2005), the Lacroix model (Cinquin et al., 2006), and TIM-2 (Minekus et al., 1999) are composed by two or more chambers connected by vessels or membranes that simulate how the lower or complete digestive tract allows the continuous flux of fluids. These systems thus reproduce the main parameters of human digestion as accurately as possible, and the experimental conditions can be tuned finely, and often, under computer control (Cordonnier et al., 2015). However, such sophisticated systems require not only complex and expensive equipment, not always available on the market, but also long times to set up the experimental conditions, as well and a high a level of expertise and competence not commonly found in all laboratories.

In the present study, the plastic transwell permeable support was used to address the issue of providing an anaerobic atmosphere to the luminal gut microbiota, and an aerobic environment to the intestinal mucosa, using semi-batch working conditions and two independent compartments. Transwell devices are well-known systems, commonly used for *in vitro* tests to simulate the intestinal absorption of drugs and other substances, included nanoparticles (Hilgers et al., 1990; Williams et al., 2016), and to grow biofilm under different physiological conditions (Standar et al., 2010; Kim et al., 2012; Wu et al., 2015; Wang et al., 2016). Compared to batch static models, transwell systems have the additional advantages of fine control of experimental conditions in different compartments, constant nutrient support and the possibility to collect the metabolites of cultured cells in long term experiments. Moreover, in contrast to dynamic systems, they are cheap, widely available on the market, easy to use, do not require a high expertise, and simplify biofilm formation and quantification, making experiments from among different laboratories more comparable. Thus, the proposed model offers the advantage of a flexible workflow, allowing independent studies in compartements with different atmosphere conditions, i.e., aerobic conditions for Caco-2 cells and anaerobic ones for human gut biofilm. Despite the precise choice to use *E. coli* as representative of the intestinal microbiota, the present system can be adapted to host different microorganisms, including Bacteroidetes and Firmicutes. We would like to point out that the designed laboratory model system is not intended to be a miniaturized version of human gut ecosystem. Rather, the purpose of the developed laboratory model system was to simplify the complexity of the intestinal ecosystem, so that it can be more easily understood. If we cannot accurately predict the behavior of a simplified laboratory system, it is unlikely we can understand enough to make predictions of natural systems (Jessup et al., 2004).

The influence of anoxic conditions on biofilm response against AgNP exposure was also studied. According to the literature, AgNPs display different properties without and with oxygen (Yang et al., 2013). Thus, it is reasonable to expect that NPs in an anaerobic environment might impact biofilm behavior differently from aerobic systems. It is also worth mentioning that, despite the fact that the fate of AgNPs on microorganisms in aerobic atmosphere has been studied to some degree, bacterial response to AgNPs under anaerobic conditions has been far less investigated, and is less understood (Gitipour et al., 2016). In line with these considerations, we performed experiments simultaneously in anaerobic and aerobic conditions and the effect of the lack/presence of oxygen in AgNP response was assessed.

In anaerobic conditions *E. coli* biofilm behavior differed, depending on the type of AgNP treatment. Indeed, when grown in the presence of AgNPs, the biofilm, for the first 72 h, showed a 2.3 ± 0.3 fold increase in viable cells compared to the control, though no reduction in oxidative stress was recorded. Indeed, the promotion of biofilm formation by exposure to sub-lethal levels of AgNPs has already been reported (Königs et al., 2015; Yang and Alvarez, 2015). This has led us, the authors of this manuscript, to speculate that stimulation of biofilm growth is an adaptive response to face the persistent presence of AgNPs, the bacteria responding by adopting a more efficient biofilm lifestyle. The stimulation of biofilm formation as a way to tolerate stress is a well-known adaptive behavior. Indeed, the presence of sub-lethal low concentrations of several antibiotics and chemicals has been shown to induce biofilm formation in a variety of pathogenic bacterial species, enabling such species to continue to exist and resist antibiotic or chemical treatment (Perrin et al., 2009; Kaplan, 2011; Machado et al., 2011).

After the 72 h period of treatment, the oxidative stress level within the biofilm appeared much increased, though the number of viable cells within the treated biofilm was still comparable with that of the control. It is reported that AgNPs can enter cells, inactivating cellular enzymes and generating endogenous ROS by interaction with biomolecules (Yang et al., 2013; Ikuma et al., 2015; Königs et al., 2015; Yang and Alvarez, 2015; Qayyum and Khan, 2016). Furthermore, it is reported that cells use ROS as a signal or cue to adapt to a changing environment (Čáp et al., 2012). Indeed, a connection between biofilm formation and oxidative stress has already been observed (Kaplan, 2011; Čáp et al., 2012; Villa et al., 2012; Cattò et al., 2015, 2017; Jakubowski and Walkowiak, 2015; Gambino and Cappitelli, 2016).

However, when AgNPs were used to treat established biofilm the outcome was different. After adding the AgNPs there was, together with increased ROS levels, the cessation of biofilm development, suggesting the occurrence of a strong cellular oxidative imbalance that the bacteria were unable to overcome efficiently in 24 h, e.g., the activation of drug resistance sessile mechanisms. As reported in recent work published by our research group, 1 μg/mL does not affect *E. coli* viability or its planktonic growth (Garuglieri et al., 2016). Thus, the data suggest an anti-biofilm mechanism subtler than a simple killing activity. Since a 1 μg/mL AgNP concentration does not affect bacterial life, the best microbial strategy might be to escape from adverse conditions, and not to persist in maintaining a biofilm lifestyle (Villa et al., 2010, 2012; Cattò et al., 2017).

It has been reported that biofilm simultaneously harbors cells in multiple states (e.g., growing, stress-adapted, dormant, inactive) and such a specific physiological status in microbial cells is an important contributing factor to antimicrobial tolerance (Williamson et al., 2012; Stewart, 2015). We found that both treated and untreated biofilm consisted of a predominant spatially distinct subpopulation of GFP-positive cells actively synthesizing new proteins. Indeed, the controls displayed a greater number of active growing cells at the air-biofilm interface than in the deeper biofilm regions where the cells were by far fewer and with very slow growth. Indeed, previous research on *Pseudomonas aeruginosa* reported the same pattern distribution, and showed that cells at the top of biofilm generally have a high mRNA abundance for genes involved in general metabolic functions, while the mRNA levels for these housekeeping genes are low in cells at the bottom of biofilm (Williamson et al., 2012). In biofilm grown for 96 h with AgNPs, the pattern distribution appeared completely inverted, with the inactive portion of cells on the top, at the interface with air, and the active one at the bottom of the biofilm, exactly where AgNP exposure took place. Indeed, a metabolically active status allows bacteria to sense their environment and actively respond to the presence of a stress factor (Stewart et al., 2015).

When the AgNP treatment involved 72 h-old established biofilm, the cells displayed a distribution pattern similar to the control. These results corroborate the hypothesis that, at this stage, where oxidative stress is far too high, the cells activate alternative defense responses rather than adaptive mechanisms to survive in the sessile life mode.

The results indicate that, in the presence of oxygen, the AgNPs only weakly influenced the biofilm, whereas the combination of oxygen restriction and AgNPs strongly modified the biofilm development. The situation was even worse when the AgNP treatment involved an anaerobic established biofilm. It has been reported that the anaerobic *E. coli* MG1655 is slow growing and patchy compared to aerobic biofilm, but some features, like the production of extracellular polymeric substances, remain unchanged (Bayramoglu et al., 2017). A closer inspection of the mRNA data reveals that essential cell processes are attenuated in anaerobic biofilm, including protein synthesis, information transfer, cell structure, regulation, and transport, suggesting that the lack of oxygen imposes severe stress on mature biofilm and this could affect some important adaptive mechanisms (Bayramoglu et al., 2017).

Our results, obtained with the *in vitro* model set up in this study, sustain a protective effect of biofilm against the genotoxic effect exerted by a 1 μg/mL AgNP concentration on Caco-2 cells. Nevertheless, at 72 h the results showed an increase in DNA damage regardless of AgNP presence (C72 and NP72): damage that can be attributed to unidentified metabolites produced by the bacterial biofilm and able to exert cell genotoxicity.

Several studies have reported the induction of oxidative stress as a conceivable molecular mechanism of AgNP toxicity (Dubey et al., 2015). However, the *in vitro* toxicological studies present in the literature suggest that many factors could influence the results, such as AgNP properties (size, concentration, type of surface-coating) and exposure protocols (cell line, cells in adhesion or suspension, state of confluency and differentiation, type of medium, time of exposure) (Zhang et al., 2016b). However, the notable differences found in the *in vitro* experimental protocols makes it difficult to compare the results obtained in our work. Let us consider only the studies on Caco-2 cells exposed to a comparable range of AgNP concentrations: Sahu et al. (2014b) demonstrated that 20 nm AgNPs decreased the viability of Caco-2 cells, starting from 10 μg/mL, but was not able to exert a genotoxic effect, estimated as presence of micronuclei by flow cytometry, up to 15 μg/mL. However, in a previous study by the same research group, the dsDNA, a measure of cell proliferation and indicative of cellular DNA damage, was significantly reduced starting from 1 μg/mL (Sahu et al., 2014a). Recently, Chen et al. (2016) focused their interests on the toxicological effects of low concentrations of AgNPs (0.7–2 μg/mL) in Caco-2 cells. Their results led them to conclude that, when Caco-2 cells were exposed to 1 μg/mL AgNPs for 24 h, intracellular ROS levels increased significantly, while the mitochondrial membrane potential that indicates mitochondrial damage appeared seriously reduced. Moreover, at 1 μg/mL AgNPs the cell membranes appeared damaged and the NPs began to induce apoptosis (Chen et al., 2016). In accordance with those studies, the present data sustain the lack of a cytotoxic effect of 1 μg/mL AgNPs, as observed by the cell viability of TNP vs. TSB, when determining the significant increase in induced DNA oxidative damage. Using the proposed experimental gut model, the preliminary interaction of 1 μg/mL AgNP concentration with biofilm significantly reduced the genotoxicity of the basolateral media to levels consistent with the basolateral collected from the control biofilm (NP24 vs. C24 and NP48 vs. C48, *p* > 0.05).

We have come to the conclusion that the results obtained with the *in vitro*, simplified model set up in this study clearly sustain a protective biofilm effect against the genotoxic effect exerted by a concentration of 1 μg/mL of AgNPs (TNP). Future work will be devoted to deeply investigate the protective role of complex multi-species biofilms on intestinal epithelial cells.

Overall, the obtained data raise potential safety concerns associated with the use of AgNPs in agri-food applications. Despite Caco-2 cells not being significantly affected by AgNPs after bacteria mediation, *E. coli* biofilm appeared to be seriously impacted when treated with AgNPs in anaerobic conditions. Earlier experiments have revealed that environmentally relevant concentrations of NPs affect short fatty acid production, hydrophobicity, sugar content of the extracellular matrix and electrophoretic mobility of the intestinal microbiota, leading to changes in the microbiota community stability (Taylor et al., 2015). Deviations in the intestinal microbiome have been associated with dozens of diseases, varying from inflammatory bowel disease to diabetes and colorectal cancer (De Vos, 2015). In this research, the important role of biofilm in protecting epithelial cells from AgNP genotoxic effects has been demonstrated. It is likely that changes in the microbial ecosystem could leave intestinal cells more exposed to negative genotoxic stressors ingested with food, e.g., AgNPs, thus increasing the level of cellular DNA oxidative damage and creating a potential for serious AgNP associated diseases.

## Author Contributions

EG and EM performed the experiments and wrote the manuscript. CC and FV conceived the experiments, participated in discussions, and improved the manuscript. FC and DE coordinated the collaboration of the authors. All authors read and approved the final manuscript.

## Conflict of Interest Statement

The authors declare that the research was conducted in the absence of any commercial or financial relationships that could be construed as a potential conflict of interest.
